# Extracellular proteolysis in structural and functional plasticity of mossy fiber synapses in hippocampus

**DOI:** 10.3389/fncel.2015.00427

**Published:** 2015-11-04

**Authors:** Grzegorz Wiera, Jerzy W. Mozrzymas

**Affiliations:** ^1^Department of Animal Molecular Physiology, Institute of Experimental Biology, Wroclaw UniversityWroclaw, Poland; ^2^Laboratory of Neuroscience, Department of Biophysics, Wroclaw Medical UniversityWroclaw, Poland

**Keywords:** MMP, tPA, mossy fiber, synaptic plasticity, LTP, CA3, ADAM, BACE1

## Abstract

Brain is continuously altered in response to experience and environmental changes. One of the underlying mechanisms is synaptic plasticity, which is manifested by modification of synapse structure and function. It is becoming clear that regulated extracellular proteolysis plays a pivotal role in the structural and functional remodeling of synapses during brain development, learning and memory formation. Clearly, plasticity mechanisms may substantially differ between projections. Mossy fiber synapses onto CA3 pyramidal cells display several unique functional features, including pronounced short-term facilitation, a presynaptically expressed long-term potentiation (LTP) that is independent of NMDAR activation, and NMDA-dependent metaplasticity. Moreover, structural plasticity at mossy fiber synapses ranges from the reorganization of projection topology after hippocampus-dependent learning, through intrinsically different dynamic properties of synaptic boutons to pre- and postsynaptic structural changes accompanying LTP induction. Although concomitant functional and structural plasticity in this pathway strongly suggests a role of extracellular proteolysis, its impact only starts to be investigated in this projection. In the present report, we review the role of extracellular proteolysis in various aspects of synaptic plasticity in hippocampal mossy fiber synapses. A growing body of evidence demonstrates that among perisynaptic proteases, tissue plasminogen activator (tPA)/plasmin system, β-site amyloid precursor protein-cleaving enzyme 1 (BACE1) and metalloproteinases play a crucial role in shaping plastic changes in this projection. We discuss recent advances and emerging hypotheses on the roles of proteases in mechanisms underlying mossy fiber target specific synaptic plasticity and memory formation.

## Introduction: Extracellular Proteolysis in Neuronal Plasticity

Activity-dependent plasticity of synaptic transmission plays a crucial role in memory encoding and in the development of the nervous system. Functional plasticity is typically accompanied by structural alterations of synapses and therefore, their morphological changes are considered a manifestation of memory trace formation (Caroni et al., 2012). Moreover, most manipulations that block structural plasticity also impair long-term plasticity and memory. Over the last decades, accumulating evidence has revealed that both neuronal function and morphology can be potently regulated by extracellular matrix (ECM) which itself shows substantial plasticity (Mukhina et al., 2012; Levy et al., 2014). Proteolytic cleavage of ECM proteins provides thus potent mechanisms to modulate the network activity and to refine neuronal circuits (Lee et al., 2008; Wlodarczyk et al., 2011). Indeed, spatially and temporally limited proteolysis of perisynaptic ECM components and membrane adhesion proteins is crucial for memory related reorganization of synaptic compartments and adjustment of signaling pathways occurring upon induction of plastic changes (Sonderegger and Matsumoto-Miyai, 2014).

The emerging pivotal role of extracellular proteolysis in neuroplasticity phenomena and cognitive tasks has provided a strong drive towards investigations into the underlying subcellular, structural and molecular mechanisms. A variety of proteases and their substrates have been found in the extracellular space in different brain regions such as cerebellum, hippocampus, cortex and amygdala. Moreover, for some proteases and respective substrates, clear correlations between their activity and experience-induced plasticity or cognitive processes have been observed (Bajor and Kaczmarek, 2013; Sonderegger and Matsumoto-Miyai, 2014). Importantly, the use of pharmacological tools as well as of transgenic animals clearly indicated that plasticity phenomena, in various neuronal pathways, may critically depend on the activity of proteases (Hirata et al., 2001; Nagy et al., 2006; Li et al., 2010b; Almonte et al., 2013). However, the major difficulty in deciphering the role of any molecular player in the plasticity phenomena is that underlying molecular signaling typically shows a high degree of pathway to pathway variability even within the same area. For instance, as it will be discussed below in detail, classic CA3-CA1 and mossy fiber–CA3 hippocampal projections show profoundly different long-term potentiation (LTP) mechanisms but in both cases LTP maintenance strongly depends on metalloproteinases (Meighan et al., 2007; Wiera et al., 2013). This points to, on one hand, a widespread presence and universal role of proteases in the neuronal plasticity but, on the other, it should be expected that the mechanisms of their involvement in shaping the plasticity are diversified among different pathways. Moreover, a closer look at the local neuronal circuits revealed that even the synapses originating from the same presynaptic axon may deeply differ in plasticity mechanisms depending on e.g., identity of postsynaptic neurons (Toth et al., 2000; McBain, 2008). The multiplicity of proteases, complexity of neuronal pathways and diversity of synaptic connections pose the major challenge in studies into the roles of proteases in the neuroplasticity phenomena and for this reason the underlying mechanisms remain poorly understood. It seems thus that the optimal strategy to study the role of proteases in the neuronal plasticity is to focus on a specific pathway and to try to correlate its physiological properties with the impact of up- and down-stream molecular players. Mossy fiber-CA3 projection in the hippocampus has been implicated in important cognitive functions including novelty detection, pattern completion and partially in pattern separation (Yassa and Stark, 2011; Kesner, 2013) and is known to be characterized by unique structural and functional plasticity (Evstratova and Tóth, 2014). In particular, extensive activity-dependent structural alterations at the level of projection topography and microstructure of different MF synapses make this pathway particularly interesting in the context of the extracellular proteolysis. Herein, we aim at providing a concise overview on the roles of extracellular proteases in functional and structural plasticity at the hippocampal mossy fiber synapses. To this end, we first present the most important features of this pathway, including its morphology and function with a particular emphasis on functional and structural plasticity, and, on this background, we review the role of the extracellular proteolysis in this projection. In particular, we discuss the impact of metalloproteinases (MMP) from metzincin family, tissue plasminogen activator (tPA), plasmin and β-site amyloid precursor protein-cleaving enzyme 1 (BACE1) and describe some recognized and putative substrates of plasticity-related extracellular proteases, which are abundantly expressed in mossy fiber terminal field. Although this review will be focused on the MF-CA3 pathway, for the sake of comparison, results obtained on the role of proteases in other, so far more extensively studied pathways (such as e.g., CA3-CA1) will be also mentioned.

## The Mossy Fiber Projection in Hippocmapus

**Figure 1:**
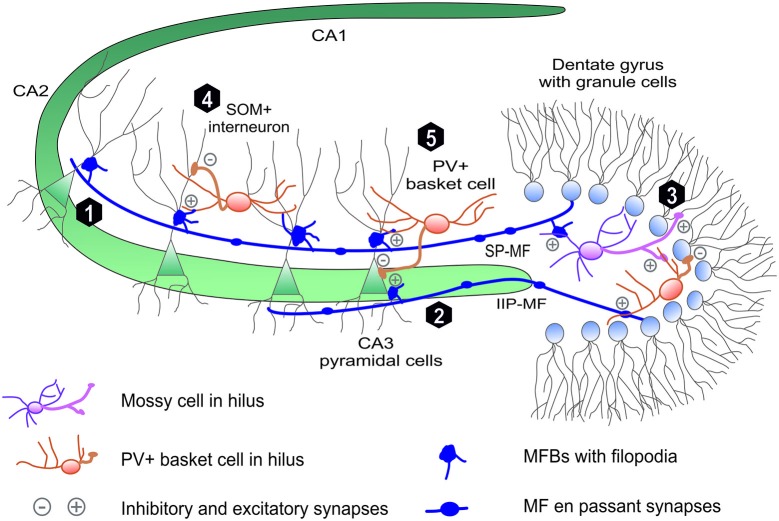
**Mossy fiber projection in hippocmapus.** The mossy fiber projection consists of axons of the dentate gyrus (DG) granule cells. Mossy fiber axons project through (1) suprapyramidal bundle (SP-MF) in stratum lucidum as well as (2) intra- and infrapyramidal projection (IIP-MF) located mainly in stratum oriens. In CA3 and hilus MFs form three types of synapses: on pyramidal cells via mossy fiber butones and on local internurons via filopodia and en passant synapses. In hillus (3) MF axons synapse on excitatory mossy cells, which backpropagate excitation to granule cells. Additionally mossy fibers directly or via mossy cells excite local hilar parvalbumin-positive internurons (basket cells) that provide feedback inhibiton to granule cells mainly in distinct lamella across the septohippocampal axis (not shown). Mossy fibers in CA3 excite local interneurons that are responsible for feedforward inhibition of CA3 pyramidal cells. These local interneuurons (e.g., PV-positive basket cells) synapse on (5) PC soma or (4) PC denditic tree (e.g., somatostatin-positive interneurons).

The mossy fiber input to CA3 region is a part of the classical trisynaptic hippocampal circuit: from the entorhinal cortex (EC) to the dentate gyrus (DG) from which granule cells project their axons (mossy fibers) to CA3 which sends Schaffer collaterals to CA1 which, in turn, sends the projection to EC through the subiculum. Dentate granule cells receive a strong polymodal input, which conveys densely coded information from the EC and participate in its conversion into relatively sparse and highly specific code transferred into the CA3 region where it undergoes associations (Witter, 2007; Kesner, 2013; Evstratova and Tóth, 2014). To perform the function of an efficient high-pass filter and, at the same time to encode a faithful information, mossy fibers developed unique anatomical and functional features. The term “mossy fibers” was coined by Ramon y Cajal who observed numerous appendages (boutons) on MF axons, which gave these fibers appearance of being covered by moss (Ramon y Cajal, 1911). Unmyelinated mossy fibers travel through the hilus where they strongly branch out and enter hippocampal CA3 region where, as a dense axonal bundle, extend parallelly to the pyramidal cell layer in stratum lucidum (Witter and Amaral, 2004; Figure 1).

The most peculiar feature of MFs is that each MF axon forms three types of glutamatergic presynaptic terminals with characteristic morphology and dissimilar functions (Figure 1). Giant mossy fiber boutons (MFBs, 3–10 μm in diameter), the hallmark of MF projection, form synaptic contacts on hilar mossy cells (7–12 per one axon) and on proximal dendrites of the CA3 pyramidal cells (MF-PC projection, 11–15 boutons per one axon; Acsády et al., 1998). Moreover, mossy fibers form also elongated filopodia protruding from MFBs and en-passant terminals both innervating local inhibitory interneurons (MF-INT, approximately 150 in hilus and 50 in CA3 region). MF passing through the hilus excite hilar mossy cells that, in turn, provide granule cells with a direct feedback excitation (Jinde et al., 2013). Additionally, mossy fibers directly or via mossy cells excite local hilar GABA-ergic interneurons (e.g., PV+ basket cells) that ensure strong feedback inhibition of granule cells (Jinde et al., 2012). In the CA3 area, mossy fibers run in two main bundles (Figure 1), the main suprapyramidal projection in stratum lucidum and the intra- and infrapyramidal projection (IIP) that runs first within the proximal extent of stratum pyramidale and startum oriens, to cross over to stratum lucidum in the CA3a region (Schwegler and Crusio, 1995). As it will be described in more detail in next sections, IIP axons in CA3 show high degree of plasticity which correlates with various forms of hippocampus-dependent learning. CA3 pyramidal neurons make synaptic contacts with MFBs via specialized complex of clustered spines called thorny excrescences (Gonzales et al., 2001). In rat brain, on average, each of 300,000 CA3 pyramidal cells is contacted by ~50 different MFBs originating from distinct mossy fiber axons (there are ca. 10^6^ granule cells in the rat DG), which indicates a convergence of neuronal information (Henze et al., 2000).

Large MF boutons in stratum lucidum are characterized by several unique morphological properties which underlie their functioning, including the ability to undergo the plastic changes. Adult mossy fibers, together with glutamate, release numerous co-transmitters like adenosine (Kukley et al., 2005), Zn^2+^ ions (Pan et al., 2011) and neuropeptides (Jaffe and Gutiérrez, 2007). The single giant MFB contains a large number of synaptic vesicles (up to 25000) with 1400–5700 vesicles as a readily releasable pool (Hallermann et al., 2003; Rollenhagen et al., 2007). Typical MFB contains from 18 to 45 separate but closely spaced active zones (Rollenhagen and Lübke, 2010; Wilke et al., 2013). Due to a small distance between individual release sites within a single MFB, the impacts of glutamate released from neighboring active zones may sum up and a crosstalk between calcium signals may take place presynaptically. Thus, even if single MF-PC active zone is characterized by a low release probability (0.01–0.05; Jonas et al., 1993), the unitary excitatory postsynaptic potentials may show a high amplitude (~10 mV) due to the numerous active zones in single MFB (Bischofberger et al., 2006; Evstratova and Tóth, 2014). Finally, close distance of the MFBs to the soma of the CA3 pyramidal cells assures minimal electrotonic dissipation of this input. Collectively, anatomical and functional studies demonstrate that MF-CA3 projection mediates a powerful monosynaptic excitatory input onto CA3 pyramidal neurons through MFBs and a disynaptic feedforward inhibition through MF-INT synapses (Figure 1). Importantly, the latter one fine-tunes recruitment of small neuronal memory-related ensembles in the CA3 field during learning (Ruediger et al., 2011).

## Functional Plasticity Hippocampal Mossy Fiber Synapses

One of the most characteristic features of the MF-PC synapses in CA3 region is strong short-term plasticity (STP). Increase in the frequency of action potentials leads to up 10-fold increase in the postsynaptic EPSP amplitude. It has been reported that intra-bouton accumulation of residual calcium during stimulation within a short time window transiently increase release probability in MF-PC synapses and drives frequency facilitation (Regehr et al., 1994; Evstratova and Tóth, 2014). As a result, even a single MF, when excited with a high frequency, is capable to trigger an action potential in CA3 pyramidal cells *in vivo* (Henze et al., 2002). In contrast, high-frequency stimulation at the CA3 MF-INT synapse leads either to relatively small frequency facilitation or even to a short-term depression (Toth et al., 2000). Since a single MF axon in CA3 forms more than ten times more synapses onto interneurons than on PC, during the low-frequency transmission, potent feedforward inhibition blocks further signal relay to CA3. Conversely, strong frequency facilitation at MF-PC synapses counterbalances powerful feedforward inhibition and efficiently activates the CA3 pyramidal cells in case of MF high frequency bursts of activity (Urban et al., 2001; Lawrence and McBain, 2003). Therefore, due to such a specific balance between strong frequency facilitation in MF-PC synapses and feedforward inhibition, MF-CA3 projection is often referred to as a conditional detonator (Urban et al., 2001). Recently, strong evidence was reported, that loose coupling of Ca^2+^ channels to Ca^2+^ sensors in MFBs together with endogenous calcium buffers with limited capacity underlie conditional detonator function of MF-PC synapses (Vyleta and Jonas, 2014). These properties enable MF-CA3 pathway to constantly change its input-output relationship as a function of granule cell spiking frequency.

The overall mean firing rate of granule cells is low, although, during hippocampus-dependent learning they may discharge high-frequency spike packages (Mistry et al., 2011) and it was found that high-frequency stimulation of MF projection induces LTP at MF-PC synapses both *in vivo* and *in vitro* (Zalutsky and Nicoll, 1990; Gundlfinger et al., 2010). It is generally accepted that the induction of LTP at MF-PC synapses is independent of NMDA receptors and expressed presynaptically as increased probability of neurotransmitter release (Tong et al., 1996; Reid et al., 2004; Nicoll and Schmitz, 2005) although NMDAR-dependent, postsynaptically expressed LTP in this pathway was also reported (Kwon and Castillo, 2008; Rebola et al., 2008). The mechanism of LTP induction at MFB requires local increase in Ca^2+^ concentration, activation of calcium/calmodulin-sensitive adenyl cyclase, protein kinase A (PKA) and protein kinase C (PKC) and subsequent phosphorylation of proteins associated with the machinery of neurotransmitter release. Four proteins were found to be essential for MF-PC LTP: small GTPase Rab3A and synaptotagmin-12, both located on synaptic vesicles and RIM1α with Munc13–1 both present in the active zone (Castillo et al., 1997, 2002; Yang and Calakos, 2011; Kaeser-Woo et al., 2013). It appears that cAMP-dependent phosphorylation of synaptotagmin-12 and interaction between RIM1α, Munc13-1 and Rab3A is required for MF-PC LTP (Kaeser et al., 2008). Additionally, presynaptic activation of the ERK and PKC signaling pathways plays a role in the activity-dependent modulation of MF synaptic vesicle mobilization and neurotransmitter release (Son et al., 1996; Vara et al., 2009). Interestingly, MF–mossy cell synapses exhibit both long- and STP that are similar to those described at MF-PC synapses (Lysetskiy et al., 2005).

Although the major expression mechanism of LTP in MF-PC synapses is presynaptic, some reports suggest involvement of the postsynaptic compartment. In this context, attention is drawn to the following factors: (1) feedback retrograde signaling from postsynaptic adhesion receptor EphB2 to presynaptic ephrin-B (Contractor et al., 2002; Armstrong et al., 2006); (2) tuning of presynaptic calcium influx and plasticity by arachidonic acid released in activity-dependent manner from postsynaptic cell membrane, which modulates voltage-gated potassium channels in MFB leading to axon potential broadening (Geiger and Jonas, 2000; Carta et al., 2014); and (3) zinc ions released as a co-neurotransmitter with glutamate from MFB which activate postsynaptic TrkB receptor in a Src kinase-dependent manner (Huang et al., 2008). Additionally, synaptic zinc also inhibits expression of postsynaptic LTP in MF-PC synapses (Pan et al., 2011).

While MF-PC synapses express NMDAR-independent LTP and LTD, ultrastructural studies have shown that NMDARs are nonetheless present at these synapses (Berg et al., 2013) and can mediate substantial postsynaptic current (Jonas et al., 1993; Kwon and Castillo, 2008). Interestingly, co-activation of synaptic NMDAR, mGluR5 and adenosine A_2A_ receptors, together with a rise in postsynaptic calcium, Src and PKC activity lead to selective enhancement of NMDAR-mediated transmission in MF-PC projection (Kwon and Castillo, 2008; Rebola et al., 2008). Moreover, this LTP of NMDARs currents acts as a metaplastic switch allowing MF-PC synapses to express classical postsynaptic NMDAR-dependent LTP of AMPA-ergic transmission (Rebola et al., 2011). Additionally, recently, it was shown that NMDAR-mediated transmission in MF-PC plays a role in heterosynaptic metaplasticity that affects plasticity in upstream CA3-CA3 synapses (Hunt et al., 2013).

I addition to the fact that mossy fibers form different synapses on various cell types, it has been demonstrated that the direction of long-term synaptic plasticity and the loci of its expression also depend on the type of postsynaptic neurons. Contrary to MF-PC, at MF-INT synapses, high-frequency stimulation leads to two types of long-term depression (Maccaferri et al., 1998). Synapses expressing AMPA receptors impermeable to Ca^2+^ ions exhibit postsynaptic and NMDAR-dependent LTD (Lei and McBain, 2002, 2004). In contrast, MF-INT synapses containing Ca^2+^ permeable AMPA receptors show presynaptic LTD in the form of reduced neurotransmitter release probability (Lei and McBain, 2004). Induction of this type of LTD requires activation of postsynaptic AMPA and presynaptic mGluR7 receptors, generation of retrograde messenger, that, in turn, causes a reduction of Ca^2+^ influx into the presynaptic filopodium (Pelkey et al., 2005, 2008).

Homeostatic plasticity (in contrast to associative, input specific and Hebbian long-term plasticity) involves cell-wide adjustments in synaptic strength. Recently, Lee et al. (2013) have shown that hippocampal MF-CA3 projection in adult animals reveals a substantial homeostatic plasticity. Indeed, MF-PC synapses undergo selective, independent and bidirectional homeostatic alterations in response to chronic changes in network activity. Homeostatic scaling in MF-PC synapses can act as a superior gain control mechanism, that modules overall network activity, leaving information encoded in remaining hippocampal synapses intact (Chater and Goda, 2013; Lee et al., 2013).

## Structural Plasticity of Hippocampal Mossy Fiber Projection and Synapses

Over the last decade, compelling evidence has indicated that hippocampal mossy fiber projection both in juvenile and adult rodents shows substantial structural plasticity at the broad range of anatomical scales—from modification of a gross axonal MF topography through changes in synapse number to remodeling of MFB ultrastructure. Using Timm’s staining method, it has been demonstrated that learning can induce a remodeling of the mossy fiber circuitry. For example, learning in Morris water maze resulted in increased spatial expansions mossy fiber axons in the CA3 stratum oriens (Ramirez-Amaya et al., 1999; Holahan et al., 2006; McGonigal et al., 2012). Extensive training in this paradigm led to increase length of infrapyramidal bundle of mossy fibers which persisted for at least 30 days (Ramirez Amaya et al., 1999). Additionally, in rats, the extent of mossy fiber IIP projection (Figure 1) correlates with acquisition of spatial memory in water maze or radial maze (Lipp et al., 1988; Schwegler and Crusio, 1995; Pleskacheva et al., 2000). Consistent with these data, induction of LTP in DG *in vivo* enlarged IIF-MF projection (Adams et al., 1997). Interestingly, MF synapses on the basal dendrites in CA3 pyramidal cell are located even closer to the soma and axon-initial segment than MFBs in the stratum lucidum (Gonzales et al., 2001). This places IIP-MF projection in a strategic position to influence action potential generation in CA3 pyramidal cells (Carnevale et al., 1997).

**Figure 2:**
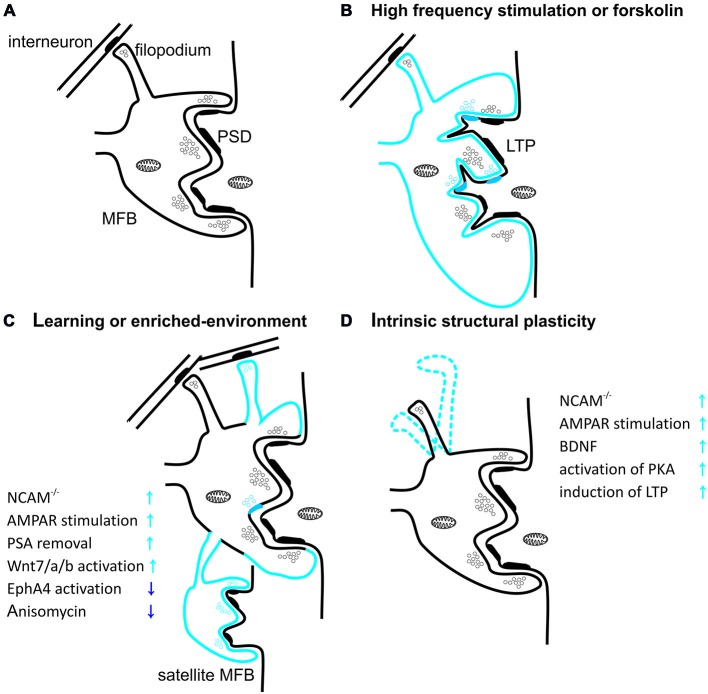
**Structural plasticity of mossy fiber presynaptic terminals. (A)** Schematic diagram showing mossy fiber bouton (MFB) contacting thorny excrescence of CA3 pyramidal cell. Thick postsynaptic lines reflect postsynaptic densities. Note that filopodial extension of mossy terminal is specialized to innervate GABAergic interneurons. **(B)** Induction of long-term potentiation (LTP) in MF-PC synapses with high-frequency stimulation or forskolin leads to increase in MFB volume and in the number of release sites per MFB. Blue color reflects structural plastic changes. Additionally, LTP promotes development of small protrusions extending from the large complex spines into the presynaptic bouton. **(C)** Spatial learning or enriched-environment promotes an expansion of mossy fibers terminals in the stratum lucidum. Furthermore, housing mice in an enriched environment leads to an increased local complexity of MFBs with frequent appearance of satellite boutons with functional release sites. Additionally hippocampus-dependent learning increases the number of filopodia protruding from MFBs and contacting feedforward projecting PV+ interneurons. **(D)** MFB filopodia are characterized by a dynamic intrinsic structural plasticity in the adult hippocampus *ex vivo*. Filopodia undergo spatial rearrangement in shape in mature hippocampal slice cultures. Plasticity processes that increase MF transmission affect also filopodia motility.

It is particularly evident that structural plasticity of the mossy fibers is expressed also at the synaptic level (Figure 2). For example, long-term stress can lead to a reduction in the density of mossy fiber synapses and to a deterioration in spatial learning performance (Sandi et al., 2003). Importantly, these impairments can be reversed through extended training for spatial tasks (Sandi et al., 2003; Scharfman and MacLusky, 2014). Conversely, enriched environment increases the number of synapses in the stratum lucidum (Tashiro et al., 2003). Furthermore, the individual MFBs differ from each other in degree of mobility and dynamics of plasticity-related structural changes. Different MFBs on the same axon show various extent of intrinsic structural plasticity, usually having one hyperplastic MFB (De Paola et al., 2003). High-frequency stimulation transforms a fraction of stable mossy fiber terminals into highly motile ones in a process involving AMPA receptors, BDNF signaling, activation of PKA and *de novo* protein synthesis (De Paola et al., 2003). In addition to diverse intrinsic motility, there is also evidence for age-related changes in MFB structural plasticity. While most hippocampal mossy fiber terminals shrink and their structural plasticity decreases with age, usually one MFB per axon gradually increases in size, suggesting the existence of “plastic hot spots” in single MF (Galimberti et al., 2006). Additionally, acquisition of a large amount of spatial information by mice housed in an enriched environment leads to increase in MFB complexity to such extent that some of them extend process with a new satellite MFB that synapse on the same or a different pyramidal cell (Galimberti et al., 2006; Gogolla et al., 2009b; Figure 2C). Moreover, the fraction of MFBs containing satellites continuously increases with mice age. It has been also reported that in animals housed in enriched environment an expansion of MFBs along apical dendrite of pyramidal cells takes place in a process that is dependent on MF-PC LTP (Gogolla et al., 2009b). Age and experience-related MFBs growth and its maintenance depends on the MF spiking activity, GABA-ergic transmission and glutamate release from MF synapses but is independent on NMDA receptors (Galimberti et al., 2006).

Recently, it was shown that also filopodia extending from MFB (contacting local interneurons) are characterized by high intrinsic mobility and prominent structural plasticity (Figure 2D). Learning was shown to be correlated with upregulation of feedforward inhibition connectivity in stratum lucidum, resulting in roughly doubling the excitatory synapses on PV-positive inhibitory interneurons (Ruediger et al., 2011). This process was implicated in increased precision of memory encoding in the hippocampus (Ruediger et al., 2012).

LTP induction in the MF-PC projection alters also the ultrastructure of pre- and postsynaptic compartment. For example, chemical LTP (induced using PKA activator—forskolin) causes an increase in the presynaptic MFB membrane surface and in the number of active zones on a single MFB (Figure 2B). MF-PC LTP was also shown to affect postsynaptic structure by increasing the number of protrusions from thorny excrescences, penetrating into the MFB (Zhao et al., 2012). These changes were found to be dependent on actin polymerization and cofilin phosphorylation (Frotscher et al., 2014).

Taken together, above described findings from distinct species and using a variety of experimental approaches clearly indicate that the local reorganization of neuronal MF circuits, synaptogenesis and modification of pre- and postsynaptic structures of MF-CA3 synapses accompany hippocampus-dependent learning and memory formation (Ruediger et al., 2012). However, the mechanisms underlying this extraordinary richness of plastic changes only start to be revealed. It needs to be reiterated that a spectacular structural plasticity of the MF-CA3 projection and synaptic ultrastructure strongly suggests involvement of extracellular proteolysis and this issue will be discussed in detail below.

## The Role of Extracellular Proteolysis in Structural and Functional Plasticity in Mossy Fiber—CA3 Pathway

In general, extracellular proteolysis may produce a variety of molecular factors involved in synaptic plasticity by means of several processes including: (1) cleavage of structural ECM constituents and membrane adhesion proteins; (2) processing and direct activation of receptors, ligands and enzymes; (3) release of ectodomains and exposure of cryptic epitopes with signaling properties; and (4) inactivation or degradation of signaling proteins or enzymes. Regulated proteolysis in the extracellular space causes changes in perisynaptic proteome and local loosening of ECM structure which may offer a permissive environment for structural plasticity (Tsien, 2013). In addition, proteolytic activation of receptors, release of its ligands or exposure of the protein-protein interaction epitopes can activate intracellular signal transduction pathways that modulate the function of synaptic receptors and expression of the plasticity-related genes in the nucleus (Levy et al., 2014). Thus proteolytic activity appears to be an ideal candidate for linking structural and functional forms of plasticity. Indeed, secretion of proteases typically depends on neuronal activity and proteolysis mediated by active enzymes affects the extracellular milieu which, in turn, triggers modulatory processes participating in the plasticity phenomena. Interestingly, in all tested hippocampal synapses, there is at least one extracellular protease that regulates their physiology.

All mammalian proteases, depending on the catalytic mechanism of peptide bond hydrolysis, are classified into five distinct classes: aspartic, threonine, cysteine, serine and metallo- proteases (listed from the smallest to the largest class in human genome; Puente et al., 2003). Here we focus on membrane-bound or soluble extracellular proteases present in mossy fiber synaptic and perisynaptic space that affect neuronal plasticity.

### Metzincins

Among extracellular proteases the most abundantly expressed in the brain are members of the metzincin family of metalloproteinases (Rivera et al., 2010). The name reflects the presence of the conserved methionine residue and catalytic zinc ion in the active site of the enzyme. Metzincin family consists of matrix metalloproteinases (MMPs), a disintegrin and metalloproteinases (ADAMs) and a disintegrin and metalloproteinases with thrombospondin motif (ADAMTSs). Over the last years, growing evidence has revealed that at least three metzincins: MMP-9, ADAM-10 and ADAM-17 regulate synaptic plasticity, learning and memory.

The MMP family includes more than 20 different genes. Depending on homology and substrate specificity, MMPs may be grouped into collagenases (MMP-1, -8, -13), stromelysins (MMP-3, -10), matrilysins (MMP-7 and only-human MMP-26), gelatinases (MMP-2, -9), transmembrane (MMP-14, -15, -16, -23, -24) or GPI-anchored (MMP-17, -25). MMPs have two major characteristics. The first is the presence of conserved cysteine residue in N-terminal propeptide sequence that maintains the enzyme in the form of an inactive zymogen (pro-MMP). The second is the HEXGHXXGXXH sequence in catalytic domain that binds zinc ion (Sekton, 2010). Activation of most MMP proteases depends on destabilization of interaction between Cys residue in prodomain with catalytic zinc. This cysteine switch occurs due to proteolytic cleavage of the prodomain that may take place extracellularly (most of MMPs) or in the lumen of endoplasmic reticulum (Furin-activated: MMP-11, -21, -28 and all transmembrane MMPs). In physiological conditions MMPs may also undergo activation through chemical modification of aforementioned cysteine in the prodomain by S-nitrosylation (e.g., by synaptically released NO) or S-glutathionylation (Vandooren et al., 2013).

### MMP-9

Over the last decade, compelling evidence has documented a particularly strong involvement of MMP-9 in neuronal physiology, learning and memory (Dziembowska and Wlodarczyk, 2012; Huntley, 2012; Stawarski et al., 2014). Indeed, MMP-9 regulates functions related to CNS development and neurogenesis (Vaillant et al., 2003; Wojcik-Stanaszek et al., 2011b), dendritic spine morphology (Michaluk et al., 2011; Sidhu et al., 2014), synaptic plasticity (Nagy et al., 2006; Wiera et al., 2013; Wojtowicz and Mozrzymas, 2014), neuronal excitability (Wójtowicz et al., 2015) and learning (Kaliszewska et al., 2012; Peixoto et al., 2012; Knapska et al., 2013; Smith et al., 2014).

**Figure 3:**
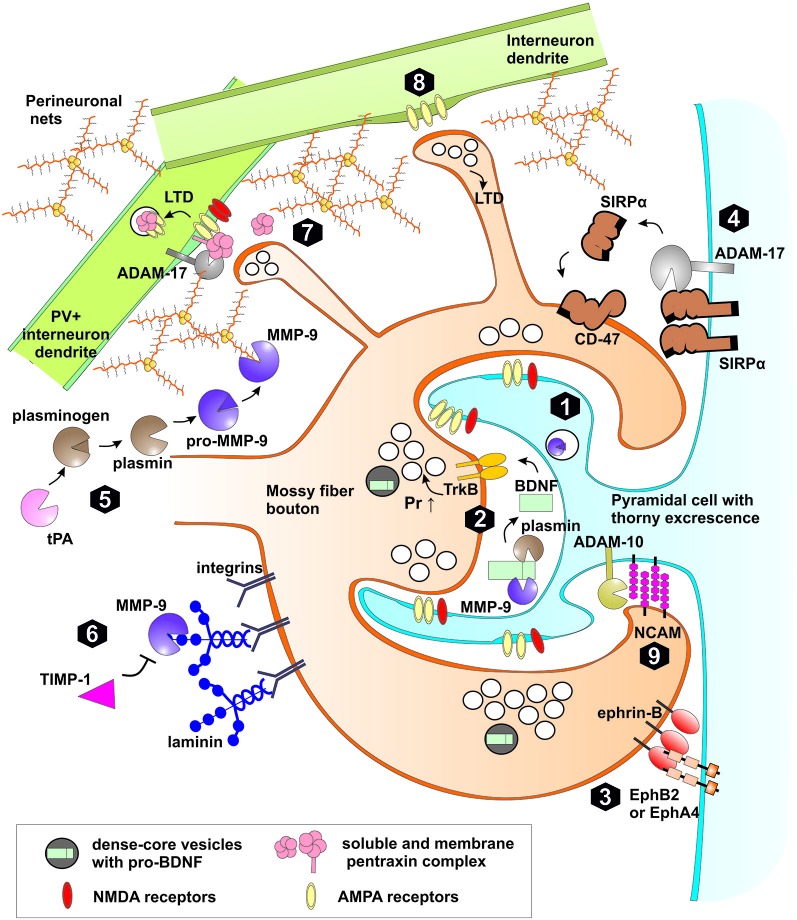
**Extracellular proteolysis in plasticity of mossy fiber synapses.** (1) High frequency stimulation leads to synthesis, release of matrix metalloproteinase (MMP)-9 and tissue plasminogen activator (tPA) to extracellular space and LTP induction in MF-CA3 synapse through increased probability of neurotransmitter release (Pr ↑). (2) MMP-9 or plasmin both activated by tPA cleaves pro-BDNF to mature form. BDNF activates TrkB receptor in the pre and postsynaptic membranes. TrkB activation affects the machinery of neurotransmitter release. (3) Trans-synaptic Eph receptor-ephrin B signaling is crucial for induction of hippocampal mossy fiber LTP. MMPs as well as neuropsin may cleave postsynaptic EphB receptors or presynaptic ephrin ligands. (4) Activity-dependent cleavage by MMPs and ADAM-17 of postsynaptic Signal regulatory protein α (SIRPα) followed by shedding of the ectodomain. The released extracellular domain of SIRPα binds to presynaptic CD47 receptor and promotes the maturation of the presynaptic terminals and LTP. (5) Activation of pro-MMP-9 in stratum lucidum requires proteolytic processing of the propeptide domain. Plasmin activated by tPA is a possible proteolytic activator of MMP-9. After activation MMP-9 and other MMPs (e.g., MMP-3) may cleave proteins of perineuronal nets (PNNs) that enwrap PV+ interneurons and restrict synaptic plasticity. (6) MMP-9 regulates mossy fiber synaptic physiology through integrin receptors by cleavage of extracellular matrix (ECM) components (e.g., laminin), release of latent integrin ligands and activation of integrin-dependent signaling pathways. Binding of endogenous inhibitor TIMP-1, secreted in neuronal activity-dependent manner terminates the activity of MMP-9 in the extracellular space. (7) Pentraxin complex composed of membrane pentraxin receptor Npr and two soluble proteins NPTX1 and NPTX2 binds and clusters synaptic AMPA receptors in excitatory synapses on interneurons. During induction of LTD, ADAM-17 cleaves Npr releasing pentraxin complex leading to AMPARs internalization. (8) In a subset of MF-INT synapses containing calcium permeable AMPA receptors and lacking NMDA receptors, presynaptic LTD can be developed in response to MF high frequency stimulation. (9) Synaptic ADAM-10 cleaves NCAM adhesion protein affecting the morphology of MFB.

Recent work showed, that presynaptic NMDA-independent LTP in the MF-CA3 projection requires MMP activity, as in the presence of a broad-spectrum MMP inhibitor (FN-439) LTP is strongly impaired (Wojtowicz and Mozrzymas, 2010). However, this inhibitor was effective only during the first 45 min after LTP induction, indicating that MMPs are critical for LTP only within a limited time window. Similar results were obtained in the CA3-CA1 pathway, where LTP is induced and expressed postsynaptically (Meighan et al., 2007). These results point to a universal role of metalloproteinases in synaptic plasticity as MMPs involvement in LTP was observed in synapses in which plasticity profoundly differs in the loci of induction/expression and NMDAR-dependence. The identity of MMP protease engaged in MF-CA3 LTP has been examined in our laboratory and it was found that in slices from MMP-9 knockout mice, LTP is impaired (Wiera et al., 2013) to a similar extent as in the presence of a broad-spectrum MMP inhibitor or a specific inhibitor of gelatinases (Wojtowicz and Mozrzymas, 2014). Interestingly, both knockdown and overexpression of MMP-9 strongly attenuated LTP maintenance showing that a fine-tuned level of MMP-9 activity is necessary for efficient LTP maintenance in the MF-CA3 pathway (Wiera et al., 2013). Importantly, treatment with exogenous active MMP-9 resulted in a stable presynaptic potentiation of MF-CA3 transmission. Additionally, in MMP-9 knockout mice, exogenous MMP-9 restored the LTP maintenance comparable to that observed in controls (Wiera et al., 2013). Thus fine-tuned MMP-9 activity appears to play a permissive role in consolidation of MF-CA3 LTP. Furthermore, LTP induction in the MF-CA3 projection is associated with increased MMP-9 (but not MMP-2) expression and activity in CA3 stratum lucidum and stratum radiatum (Wiera et al., 2012). This finding appears consistent with observation that MMP-9 mRNA is transported along microtubules to dendritic spines (Sbai et al., 2008; Dziembowska et al., 2012). Janusz et al. (2013) have shown in neuronal cultures that dendritic MMP-9 mRNA is inactive due to an association with FMRP protein and is directed to translation after synaptic activity-dependent polyadenylation (Janusz et al., 2013). MMP-9 is secreted outside the cell, as an inactive proenzyme and is subsequently activated (Murphy and Nagase, 2011; Figure 3). Thus the question arises, how MMP-9 is activated in the extracellular space of the stratum lucidum? A good candidate for this purpose could be synaptically released nitric oxide (Gu et al., 2002; O’Sullivan et al., 2014). However, block or knockdown of nitric oxide synthase does not affect the magnitude of LTP in MF-CA3 projection (Doreulee et al., 2001) arguing against this possibility. Several other mechanisms await verification. In particular, tPA/plasmin system is highly expressed in the mossy fibers and may activate MMP-9 (Tai et al., 2010; Hwang et al., 2011). Similar to MMP-9, its endogenous inhibitor TIMP-1 is also upregulated in stratum lucidum by neuronal activity (Newton et al., 2003). This raises the possibility that extracellular MMP-9 activation and its subsequent inhibition by TIMP-1 defines a specific time window for the proteolytic activity that allows for long-term plastic changes in MF synapses. These functional and pharmacological studies were paralleled by behavioral investigations which indicated that novel-object recognition, a task dependent on MF-CA3 synaptic transmission and plasticity, (Kesner, 2007; Bednarek and Caroni, 2011; Hagena and Manahan-Vaughan, 2011) is impaired in MMP-9 knockout mice (Mizoguchi et al., 2010).

MMP-9 was also found to play an important role in the structural aspect of the synaptic plasticity. Indeed, several studies demonstrate that administration of exogenous protease or MMP-9 overexpression can regulate dendritic spine shape and stability. For example, Wang et al. (2008b) have shown that acute MMP-9 application gives rise to a spine enlargement and morphological modification towards a mushroom-like phenotype in the CA1 hippocampal neurons. On the other hand, prolonged treatment of cultured hippocampal neurons with active MMP-9 tended to induce a filopodia-like phenotype of dendritic spines (Michaluk et al., 2011) which is accompanied by increased membrane mobility of NMDAR, synaptic clustering of AMPA receptors (Michaluk et al., 2009; Szepesi et al., 2014) and increased decay constant of mEPSCs (Wiera et al., 2015). These findings suggested that prolonged activity of MMP-9 gave rise to a functional and morphological “juvenilization” of dendritic spines and synaptic currents. Additionally, it was also shown that exogenous active MMP-9 induces growth of thin spine head protrusions (Szepesi et al., 2013) already described in MF-PC synapses (Zhao et al., 2012). However, so far, little is known about involvement of MMP-9 in MF structural changes after LTP and during the acquisition of hippocampus-dependent memory. Temporal lobe epileptogenesis is often accompanied by an aberrant sprouting of hippocampal mossy fibers and the formation of abnormal MF synapses with granule cells. It has been shown that blocking of the MMP activity with broad-spectrum inhibitor decreases abnormal growth of mossy fibers during pentylenetetrazole-induced kindling (Yeghiazaryan et al., 2014). Moreover, MMP-9 protein and activity were detected in DG molecular layer where sprouted MF synapses are formed and in CA3 region of epileptic hippocampus (Wilczynski et al., 2008; Takács et al., 2010; Konopka et al., 2013). Interestingly, development of seizures in kindled mice depends on the conversion of pro-BDNF to mature BDNF by MMP-9 (Mizoguchi et al., 2011). Taken together, these studies indicate that the MMP-9 activity is essential to support the synaptic modifications associated with MF-CA3 LTP and structural plasticity in hippocampus.

Proteomics experiments using mass spectrometry have shown that the number of putative MMP-9 substrates may reach up to several hundred proteins. Among them there are extracellular, cytoplasmic and nuclear proteins (Overall and Blobel, 2007; Xu et al., 2008; Butler and Overall, 2009; Cauwe et al., 2009; Cauwe and Opdenakker, 2010; Zamilpa et al., 2010). Whether a putative MMP-9 substrate is really cleaved *in vivo* depends on several factors including: (1) expression level of both protease and substrate; (2) access of the proteases to the substrate (e.g., to proteins present in the cell nucleus or in the vicinity of the cell membrane); (3) binding of a protease to the components of ECM, which defines sites of protease activity; (4) duration of time window between MMP activation and inhibition by binding of TIMP inhibitors; (5) interaction between the MMP-9 hemopexin, hinge domain or fibronectin repeats with substrate that increase the cleavage specificity. Given these limitations, only a small part of the putative MMP-9 substrates is actually proteolyzed *in vivo* giving rise to physiologically important effects. While largely untested, there are many putative MMP-9 substrates present in stratum lucidum and hilus, which after cleavage may shape structural and functional plasticity of mossy fiber synapses.

One of aforementioned MMP-9 neuronal substrate is pro-BDNF, which is secreted from mossy fiber terminals and postsynaptic cell during burst activity (Danzer and McNamara, 2004; Li et al., 2010a; Dieni et al., 2012). MMP-9 potentially cleaves pro-BDNF to produce mature BDNF that binds to and activates TrkB receptor. Thus MMP-9 may regulate the availability of active BDNF in the perisynaptic space (Ethell and Ethell, 2007; Mizoguchi et al., 2011). Moreover, putative activation of pro-BDNF released from mossy fibers by dendritic MMP-9 may act as a coincidence detector of pre-post activity during the induction of MF-PC LTP (Figure 3). NMDA receptor is one of the few known synaptic coincidence detectors of pre-post activity as it is activated by presynaptically released glutamate and postsynaptic depolarization. However, LTP in the MF-PC synapses is independent on NMDAR and is routinely induced experimentally in the presence of APV. Therefore, in the MF-PC synapses, induction of LTP may require a different coincidence detector and activation of pro-BDNF by MMP-9 is a good candidate for such a mechanism.

MMP-9 activity affects both postsynaptic NMDAR-dependent LTP in CA3-CA1 pathway and NMDAR-independent, presynaptic LTP in MF-PC synapses, raising the question how MMP-9 can regulate such different types of long-term plasticity. Early data suggested that BDNF/TrkB signaling is activated both in pre- and postsynaptic compartment (reviewed in Edelmann et al., 2014). In hippocampal CA3-CA1 synapses, BDNF augments postsynaptic transmission through activation of MAPK pathway, modulation of GluN2B-conatinnig NMDAR and induction of calcium inward currents (Minichiello, 2009). In contrast, mossy fibers express TrkB receptor that is activated by synaptic BDNF (Helgager et al., 2014). Moreover, activation of TrkB signaling enhances glutamate release targeting presynaptic proteins engaged in MF-PC LTP such as: synapsins, Rab3a and Rim1α (Jovanovic et al., 2000; Thakker-Varia et al., 2001; Zakharenko et al., 2003; Yano et al., 2006; Simsek-Duran and Lonart, 2008). These results together indicate that MMP-dependent processing of pro-BDNF and TrkB activation may be necessary for functional presynaptic plasticity and structural alteration of MF-CA3 synapses, although the latter possibility awaits thorough investigations.

Integrin adhesion receptors, a major family of ECM receptors in the brain, are involved in numerous molecular mechanisms related to synaptic plasticity, learning and memory (McGeachie et al., 2011). In particular, integrin signaling modulates morphology and intrinsic motility of dendritic spines (Levy et al., 2014). Moreover, MMP-9 involvement in structural and functional plasticity is strongly dependent on integrin signaling. Indeed, integrin β1 blocking antibody abolishes the effect of exogenous MMP-9 on NMDAR membrane diffusion (Michaluk et al., 2009), dendritic spine morphology in neuronal cultures (Wang et al., 2008b) and blocks synaptic potentiation after administration of MMP- 9 in CA3-CA1 pathway (Nagy et al., 2006). Interestingly, mossy fibers show immunoreactivity for α2-, β4- and β5-integrins (Wu and Reddy, 2012) while perisynaptic astrocyte processes in stratum lucidum contain β1-integrin (Schuster et al., 2001). Although MMP-9 cleaves directly β4-integrin *in vitro* (Pal-Ghosh et al., 2011), the present evidence indicates rather an indirect interaction. β4-subunit containing integrins present in mossy fibers are considered as laminin receptors (Srichai and Zent, 2010). MMP-9 can locally cleave perisynaptic ECM proteins (such as laminin or fibronectin), that, in turn, might reveal their RGD sequence and activate integrin signaling that could locally support activity-dependent and intrinsic structural plasticity (Levy et al., 2014). As already mentioned, some MFBs are hyperplastic and develop additional satellite boutons (Galimberti et al., 2010). Maintenance of these local hot spots of structural plasticity in single axon may require MMP-9 activity and integrin signaling (Figure 3). In hippocampal CA3-CA1 and cortical synapses, MMP-9 cleaves the membrane adhesion protein ICAM-5 that is most abundant on dendritic filopodia (Tian et al., 2007; Lonskaya et al., 2013). In neuronal cultures postsynaptic ICAM-5 interacts with presynaptic β1 integrin and blocks structural transition from filopodium to mature spine (Ning et al., 2013; Gahmberg et al., 2014). ICAM-5 cleavage promotes spine maturation and release of soluble extracellular domain of ICAM-5 increases synaptic AMPA receptor expression and cofilin phosphorylation (Lonskaya et al., 2013). Although β1-integrin is not expressed in mossy fiber synapses, the role of ICAM-5 proteolysis and activation of perisynaptic integrins in stratum lucidum await clarification.

Recently, it was shown that MMP-dependent cleavage of signal regulatory protein α (SIRPα) leads to release of extracellular SIRPα domain that binds to presynaptic CD47 receptor and promotes maturation of presynaptic terminals in cell cultures. Moreover, synaptic transmission and LTP in CA1 region is significantly impaired in SIRPα null mice (Toth et al., 2013). Interestingly, both SIRPα and CD47 are most abundant in the stratum lucidum (Matozaki et al., 2009). This raises the possibility that MF activity leads to MMP-9 secretion, shedding of SIRPα ectodomain that, in turn, regulate presynaptic MF-PC plasticity (Figure 3). It will be interesting to further elucidate the molecular determinants of MMP-dependent SIRPα proteolysis in MF synapses, and to find out how CD47 activation regulates presynaptic functions.

In addition to substrates described above, MMP-9 cleaves structural proteins that form the backbone of the excitatory synapses such as neuroligin-1 (Peixoto et al., 2012), EphB receptors (Lin et al., 2008), netrin-3 (van der Kooij et al., 2014), SynCAM-2 (Bajor et al., 2012) and β-dystroglycan that is present mainly at inhibitory synapses (Lévi et al., 2002; Michaluk et al., 2007; Ganguly et al., 2013). MMP-9 processes also short-lived proteins responsible mainly for synaptic signaling, including interleukins (Amantea et al., 2007), β-amyloid peptide (Ridnour et al., 2012) and insulin-like growth factor-binding proteins (Nishijima et al., 2010). Thus, MMP-9 proteolysis may be involved in mechanisms underlying various types of memory, characterized by a different time span. Indeed, the short-term synaptic changes are likely to be related to cleavage of proteins with fast turnover, whereas truncation of proteins with long half-lives would produce long-lasting changes which are important for LTP maintenance.

In the last decade, thanks to proteomic techniques, it was realized that the repertoire of metzincin substrates is much wider than expected and, surprisingly, a part of putative or confirmed MMP substrates reside inside the cell. Moreover, the active forms of MMP-2, -3, -9, -12 have been found in the cytosol, mitochondria, and nucleus of neurons and glia (Cauwe and Opdenakker, 2010; Mannello and Medda, 2012). Intraneuronal activity of MMPs has been reported in pathological processes such as Parkinson’s disease (Choi et al., 2011), cerebral hypoxia (Yang et al., 2010; Wojcik-Stanaszek et al., 2011b) or epilepsy (Konopka et al., 2013). Interestingly, induction of LTP in MF-CA3 pathway in slices, using high-frequency stimulation of mossy fibers, increases *in situ* MMP-9 activity in the cytoplasm of CA3 pyramidal neurons (Wiera et al., 2012). Additionally, increase in MMP-9 protein was observed in the cytoplasm and nuclei of CA3 pyramidal cells after the induction of MF LTP (Wiera et al., 2012). In the same study, nuclear MMP-2 was also reported in neurons, although its expression and activity has not changed after LTP. It seems thus that unraveling substrates of intraneuronal MMPs and mechanisms of their regulation emerges as a novel challenge in studies on MMP-9 role in neuronal physiology.

### ADAM-10 and ADAM17/Tace

Membrane-anchored proteases, belonging to the ADAM subfamily of metzincins, are able to cleave transmembrane proteins close to the surface of cell membrane in a process referred to as ectodomain shedding. Most of ADAMs are built with (from C-terminus): a cytoplasmic domain, a type I transmembrane sequence, an EGF-like domain, a cysteine-rich region, a disintegrin domain and a metalloproteinase domain (in some ADAMs inactive). Two most studied neuronal ADAMs are ADAM-10 and ADAM-17 also known as TNF-α converting enzyme TACE (Weber and Saftig, 2012). Studies on the roles of ADAM-10 and ADAM-17 in learning-related processes are hampered by the fact that ADAM-10 knockout mice are embryonic lethal and ADAM-17-deficient mice die perinatally or show increased mortality in adulthood depending on genetic background (Rivera et al., 2010). ADAM-10 and ADAM-17 are known due to their α-secretase activity responsible for APP cleavage and generation of extracellular, soluble, non-amyloidogenic sAPPα domain that is endowed with signaling functions (Saftig and Reiss, 2011). Conditional neuronal ADAM10 knockout mice show impairments in hippocampus-dependent learning, almost abolished LTP and atypical prevalence of stubby spine morphology in CA1 region (Prox et al., 2013). Additionally, constitutive overexpression of ADAM-10 negatively affects learning and memory in mice (Schmitt et al., 2006). These findings are reminiscent of the observation that LTP maintenance in MF-CA3 projection requires fine-tuned proteolytic activity mediated by MMP-9 (Wiera et al., 2013). Increased synaptic activity, through deacetylase SIRT-1 induces ADAM-10 transcription and subsequent translation (Donmez et al., 2010; Gao et al., 2010). ADAM-10 as an integral membrane protein is present in postsynaptic densities of excitatory synapses where it is trafficked due to interaction with synaptic scaffold protein SAP97 (Musardo et al., 2014). In response to synaptic stimulation ADAM-10 cleaves neuronal N-cadherin in primary hippocampal neurons (Malinverno et al., 2010). Interestingly, N-cadherin regulates mossy fiber fasciculation (Bekirov et al., 2008) and have an important role in modulating synaptogenesis, spine formation, and synaptic plasticity in CA3-CA1 projection (Arikkath and Reichardt, 2008). ADAM-10 has also been reported to cleave ephrin-A2 that forms trans-synaptic complex with EphA receptors in cell cultures (Hattori et al., 2000). Galimberti et al. (2010) have found that after disruption of EphA4 signaling in mossy fiber circuits, single MFB establishes significantly more satellite boutons, suggesting that ADAM-10-dependent proteolysis of synaptic ephrin-A may be involved in regulation of the number of MFBs in hilus and stratum lucidum.

ADAM-10 and ADAM-17 both shed many classical cadherin adhesion proteins (Weber and Saftig, 2012). One of them, cadherin-9 is expressed selectively in mossy fibers and CA3 pyramidal cells and knockout or interference with cadherin-9 adhesion severely disrupts morphology of MFBs and filopodia (Williams et al., 2011). Additionally, both ADAM-10 and ADAM-17 cleave synaptic adhesion protein NCAM in neuronal cultures (Hinkle et al., 2006). NCAM, expressed in stratum lucidum, is responsible for MF growth and lamination, but is dispensable for MF-CA3 LTP recorded in slices (Cremer et al., 1997, 1998; Bukalo et al., 2004). Nevertheless, NCAM knockout mice have more filopodia emanating from MFBs and, additionally, they are hyperdynamic and excessively branched (De Paola et al., 2003). Interestingly, polysialylated form of NCAM (PSA-NCAM) is present in stratum lucidum and affects structural plasticity of MFB (Seki and Arai, 1999; Galimberti et al., 2010). Thus, activity-dependent proteolysis of NCAM may constitute an important factor in shaping the structural plasticity of the mossy fibers projection (Figure 3).

Recent studies in cell cultures and hippocampal CA3-CA1 pathway in slices point compellingly to a role for ADAM-10 in LTD (Marcello et al., 2013). Induction of LTP in this projection decreases whereas LTD stimulates ADAM-10 synaptic localization and activity (reviewed in Musardo et al., 2014). In addition, activation of mGluR1/5 receptors in neuronal cultures induces MMP and ADAM-17-dependent cleavage of synaptic pentraxin receptor Npr, release of its extracellular domain, internalization of AMPA receptors and expression of LTD (Cho et al., 2008). As discussed above, the hallmark of MF-CA3 pathway is the opposite impact of high frequency stimulation on synaptic plasticity in MF-PC and MF-INT synapses in stratum lucidum (Figure 3). At the network level, induction of LTD in mossy fiber excitatory synapses onto PV^+^ interneurons (MF-PV^+^ INT) causes decreased recruitment of feedforward perisomatic inhibition, which affects time window for synaptic integration in CA3 pyramidal cells (McBain, 2008). Recently, Pelkey et al. (2015) have shown that neuronal pentraxins control strengthening of synapses on PV+ interneurons. There are three neuronal pentraxins: two secreted to extracellular space (NPTX1 and NPTX2 also called Narp) and one membrane neuronal pentraxin receptor (Npr). All three pentraxins form heteropentamers that are soluble or tethered to the cell membrane by Npr (Kirkpatrick et al., 2000). Neuronal pentraxins are broadly expressed in the hippocampus, cerebral cortex and cerebellum (Pribiag and Stellwagen, 2014). Moreover, NPTX1 is highly expressed by CA3 PC and, to a lesser extent, by DG granule cells (Schlimgen et al., 1995; Dodds et al., 1997). Activity-regulated protein NPTX2 (Narp) is abundant in mossy fiber terminal field (Tsui et al., 1996; Xu et al., 2003), similar to Npr (Cho et al., 2008). NPTX2 knockout mice are hypersensitive to kindling-induced seizures (Chang et al., 2010) in an analogos way as transgenic mice overexpressing MMP-9 (Wilczynski et al., 2008) and in agreement with attenuation of seizures by inhibitor blocking ADAM-17 activity (Meli et al., 2004). Additionally, both NPTX2 and Npr are enriched at excitatory synapses on PV+ interneurons where they cluster GluA4-containing AMPA receptors, increasing thereby synaptic currents (Chang et al., 2010; Gu et al., 2013; Pelkey et al., 2015). Furthermore, Npr and NPTX2 double deficient mice show impaired feedforward inhibition and increased duration of critical period plasticity in hippocampus (Pelkey et al., 2015). Thus, ADAM-17-dependent pentraxin cleavage is likely to be involved in target cell-specific synaptic plasticity in MF-INT synapses. Future studies are needed to elucidate the relationship between extracellular proteolysis, activity dependent synaptogenesis in stratum lucidum and induction of LTD in MF-INT synapses. Moreover, MMP activity and synapse-specific cleavage of Npr in MF-INT synapses may determine the local short-lasting opening of a “critical period” plasticity of excitatory transmission between principal cells in adult hippocampus.

Hippocampal neurons and glial cells express a plethora of other extracellular metalloproteinases that may also modify synaptic functions and several lines of evidence support the notion that, besides MMP-9, also other metzincins participate in certain types of synaptic plasticity. Indeed, MMP-7 affects dendritic spine morphology and NMDAR currents in primary neuronal cultures (Bilousova et al., 2006; Szklarczyk et al., 2008) and ADAMTS-4 cleaves ECM proteoglycans enhancing neuroplasticity (Lemarchant et al., 2014). Moreover, learning is associated with elevated expression of MMP-3 in rodents (Olson et al., 2008; Wright and Harding, 2009) and MMP-12 is upregulated after bicuculine-induced chemical LTP (Pinato et al., 2009). This suggests that additional metalloproteinases may regulate synaptic transmission and synaptic morphology in the mossy fiber pathway, but further studies are needed to understand their roles at MF synapses in the hippocampus.

### Serine Proteases: tPA/Plasmin System

Tissue plasminogen activator (tPA) is a serine protease that converts plasminogen to plasmin. In neurons *tpa* is an immediate-early gene and the increase in synaptic activity induces its expression (Qian et al., 1993). tPA together with plasmin modulate long-term plasticity in hippocampus, cortex and amygdala as well as learning and memory (reviewed in Samson and Medcalf, 2006; Almonte and Sweatt, 2011). tPA and plasmin are abundant in mossy fiber terminal field (Salles and Strickland, 2002; Taniguchi et al., 2011) and both are stored in dense-core vesicles and released in an activity-dependent manner from axons or dendrites (Lochner et al., 2006). Interestingly, tPA appears to play a similar role in the mossy fibers to that discussed above for MMP-9. Indeed, akin to MMP-9, blocking the activity of tPA/plasmin system or tPA knockout impairs induction of MF-CA3 LTP while exogenous active tPA enhances LTP and potentiates basal MF synaptic transmission (Huang et al., 1996; Baranes et al., 1998). Moreover, tPA knockout mice exhibit impaired object recognition memory, which is critically dependent on MF-CA3 projection and on MMP-9 activity (Benchenane et al., 2007; Kesner, 2007; Mizoguchi et al., 2010; Bednarek and Caroni, 2011). Furthermore, blocking tPA or MMP-9 activity decreases aberrant mossy fiber sprouting in epileptic hippocampus (Wu et al., 2000; Yeghiazaryan et al., 2014). These similarities between MMP-9 and tPA involvement in MF plasticity suggest their potential crosstalk. Indeed, direct or indirect activation of pro-MMP-9 by tPA/plasmin is the most plausible scenario (Chakraborti et al., 2003), but this hypothesis requires verification.

Both plasmin and MMP-9 can cleave and activate pro-BDNF to mature BDNF during the maintenance phase of CA3-CA1 LTP and epileptogenesis (Pang et al., 2004; Nagappan et al., 2009; Mizoguchi et al., 2011). Moreover, BDNF, in a feedback-manner activates TrkB and promotes transcription of *tpa* and *mmp-9* genes in neuronal culture (Kuzniewska et al., 2013; Bennett and Lagopoulos, 2014). Besides, activation of pro-BDNF and pro-MMP-9, tPA/plasmin system cleaves also GluN1, GluN2A and GluN2B subunits of NMDA receptor *in vitro*, changing its kinetic and pharmacological properties (Benchenane et al., 2007; Yuan et al., 2009; Ng et al., 2012). This raises a possibility that tPA/plasmin may be involved in NMDA-dependent metaplasticity described in MF-PC synapses (Hunt et al., 2013).

### Serine Proteases: Neuropsin

In MF-PC synapses, the postsynaptic EphB2 interaction with presynaptic ehrin-B2 builds high affinity adhesion complex, which mediates MF-PC trans-synaptic signaling (Contractor et al., 2002). EphB2 and ephrin-B2 bind to each other with a high affinity which is it unlikely to spontaneously dissociate. To “reset” the synapse to the state prior the formation of ephrin-Eph receptor complex, cell internalizes the whole complex together with a part of neighboring membrane (Zimmer et al., 2003) or may release proteases that cleave ephrin or its receptor. In the MF-CA3 and CA1-CA3 projections, interference with the EphB-ephrinB complex, using recombinant soluble ligands or genetic knockouts causes impairment of LTP and dendritic spine structural plasticity (Contractor et al., 2002; Grunwald et al., 2004; Armstrong et al., 2006; Kayser et al., 2008). It was shown in the amygdala that stress-related learning and induction of LTP is accompanied by a neuropsin-dependent cleavage of EphB2 (Attwood et al., 2011). The activity of serine protease neuropsin has a pivotal role in establishing early phase of LTP in CA3-CA1 pathway, synaptic tagging and hippocampus-dependent learning (reviewed in Shiosaka and Ishikawa, 2011; Tamura et al., 2013). Additionally, neuropsin cleaves neuregulin-1, releasing part of its extracellular domain which activates a receptor tyrosine kinase—ErbB4 in synapses onto PV-positive hippocampal interneurons (Tamura et al., 2012). In turn, ErbB4 activation strengthens GABAergic feedforward synaptic transmission (Lu et al., 2014). Further studies, including physiological, morphological and behavioral approaches are necessary to clarify whether neuropsin-dependent cleavage of EphB-ephrin complex and neuregulin-1 regulates synaptic plasticity of MF-CA3 pathway, feedforward inhibition in CA3 and experience-driven structural plasticity of mossy fiber synapses.

### Aspartate Protease: Beta-Secretase-1

BACE1 has been identified as a major neuronal β-secretase responsible for formation of β-amyloid peptide, which is thought to be responsible for the amyloid plaques formation, which is the hallmark of the Alzheimer’s disease. Importantly, BACE1 protein is abundantly expressed in the mossy fiber projection in stratum lucidum (Laird et al., 2005) and undergoes retrograde transport from dendrites to axons (Buggia-Prévot et al., 2013). In MF-PC synapses, LTP is absent in BACE1 knockout mice (Wang et al., 2008a, 2014) and this impairment may be rescued by pharmacologically induced increase in the presynaptic calcium influx (Wang et al., 2010). Augmentation of short-term plasticity (STP) in MF-PC synapses in BACE1 deficient mice suggests BACE1-dependent tuning of presynaptic function (Laird et al., 2005; Wang et al., 2008a). Moreover, regulation of release probability by BACE1 is synapse-dependent, because knockout of BACE1 protein increases STP in MF-PC, but not in MF-INT synapses (Wang et al., 2014).

Hitt et al. (2012) have indicated yet another aspect of BACE1-dependent hippocampal plasticity. BACE1-deficient mice exhibit shortened and disorganized intra- and infrapyramidal (IIP) mossy fiber projection. Moreover, during the developmental growth of IIP-MF projection, BACE1 may cleave an adhesion protein—a close homolog of L1 (CHL1) which is abundant in mossy fiber terminal filed (Hitt et al., 2012; Kuhn et al., 2012). Additionally, CHL1-deficient mice show impairments in MF-dependent novel object recognition memory (Pratte and Jamon, 2009). It remains unclear, however, whether BACE1-dependent CHL1 cleavage is involved in the experience-driven outgrowth of IIP-MF axons, MF-CA3 LTP and learning. Because CHL1 protein is expressed also by PV+ interneurons in the hippocampus, it is also possible that the BACE1 activity may regulate the connectivity of feedforward inhibition in CA3 (Nikonenko et al., 2006; Wang et al., 2014).

## Feauture Directions: Extracellular Proteolysis and Target Cell-Dependent Plasticity

A unique feature of hippocampal mossy fibers at early postnatal development is the release of GABA (Safiulina et al., 2006). The immature hippocampus is characterized by a network-driven giant depolarizing potentials, which are generated by a synergistic action of glutamate and GABA, both depolarizing at early developmental stages (Ben-Ari et al., 2007). Pairing single postsynaptic spikes with unitary MF GABA_A_R-mediated postsynaptic potentials consistently upregulates or downregulates synaptic strength according to the temporal order of stimulation. Positive pairing induces LTP which is L-type calcium channel and BDNF-dependent (Sivakumaran et al., 2009; Caiati et al., 2013). In mature hippocampus high-frequency stimulation was shown to increase the dynamics of structural changes in MF terminals (De Paola et al., 2003). The impact of juvenile mossy fiber GABA-ergic plasticity on the postnatal developmental growth of MF axons and their activity-dependent morphological changes remain to be determined. In particular, the role of neuronal activity-induced proteolysis in the extracellular space in the mossy fiber GABA-ergic plasticity (Safiulina et al., 2010) or mossy fiber developmental growth (Holahan et al., 2007; Galimberti et al., 2010) awaits elucidation. Most interestingly, in this context, the highest expression of MMP-9 and tPA in hippocampus and cortex occurs in early postnatal development (Zheng et al., 2008; Aujla and Huntley, 2014).

As already mentioned, plasticity in the MF-PC and MF-INT synapses shows an interesting property of target-cell specificity (McBain, 2008). The mechanism of this differential plasticity is not fully understood but synapse-specific interactions between ECM elements and extracellular proteases are likely candidates. Interestingly, recently, for the first time, it has been also shown that MMP-dependent degradation of ECM proteins promotes reorganization of inhibitory innervation in hippocampus during increased neuronal activity (Pollock et al., 2014). A very characteristic ECM structures in the brain are so called perineuronal nets (PNNs) which consist of aggregates of proteins and glycosaminoglycans surrounding soma, proximal dendrites and axon initial segment, mainly of PV+ interneurons (Wang and Fawcett, 2012). PNNs appear at later stages of interneuron maturation and affect their electrophysiological properties (Miyata et al., 2005; Van den Oever et al., 2010; Liu et al., 2013; Slaker et al., 2015). Administration of chondroitinase, that enzymatically digests glycosaminoglycans and disrupts PNN structure, reopens critical period plasticity in adult animals (Pizzorusso et al., 2002) as well as increases spine motility and structural plasticity in the visual cortex (de Vivo et al., 2013). It is worth emphasizing, that chondroitinase is not an endogenous protein and other neuronal enzymes like proteases and sulfotransferases are responsible for PNN remodeling (Matsui et al., 1998; Matthews et al., 2000; Miyata et al., 2005; Valenzuela et al., 2014; Rankin-Gee et al., 2015). It has been already shown that MMPs cleave almost all proteoglycans present in PNNs, e.g., aggrecan, neurocan, brevican, and phosphacan (Van Hove et al., 2012). Interestingly, expression and activity of most plasticity-related extracellular proteases peak during critical period (Zheng et al., 2008; Aujla and Huntley, 2014). Moreover, tPA activity permits synapse remodeling during ocular dominance plasticity similarly as does PNN digestion (Mataga et al., 2002; Oray et al., 2004; Gogolla et al., 2009a). Roger Tsien has recently proposed an intriguing hypothesis that very long-term memories may be stored as patterns of holes excised in the PNNs by extracellular proteases released and activated upon increased neuronal activity (Tsien, 2013). In this scenario, molecular determinants of proteolytic activity exerted upon PNNs, which embed MF-INT and inhibitory synapses, are expected to play a central role in this mechanism of memory storage. Multiplicity of important physiological mechanisms which are critically regulated by PNN remodeling underscores the importance of ECM proteolysis in modulation of neuronal network functions, especially in a long time scale. It is expected that future studies will shed more light on how crosstalk between PNNs, mossy fiber synapses and extracellular proteolysis contributes to the formation of hippocampal memory engrams.

Modification of perisynaptic environment by ECM proteolysis might give rise to yet another mechanism of synaptic signals modulation. It is known that the time course of the synaptic agonist transient is a key determinant of the synaptic currents kinetics (Mozrzymas, 2004; Barberis et al., 2011) and geometry of the synaptic cleft as well as of its nearest environment plays a crucial role in shaping the time course of synaptic agonist (e.g., Barbour et al., 1994; Cathala et al., 2005). Considering a particularly complex structure of synapses in the MF-CA3 projection this mechanism of synaptic signals regulation can be of particular importance but whether or not it is the case still awaits to be investigated.

There is compelling evidence that memory allocation to specific neurons and synapses in neuronal circuits is not random, and that specific processes, such as plastic changes in neuronal excitability and synaptic tagging, determine the exact sites where memories are stored (Rogerson et al., 2014). Precise temporal regulation of transcription and translation of new proteins are necessary for synapse strengthening and maintenance of plastic changes, in spite of the continuous protein turnover. It remains unexplained how one translation machinery in a single neuron, may be responsible for strengthening of specific individual synapses among thousands of synapses present in the dendritic tree. A growing body of evidence suggests that process of synaptic tagging and capture may explain the synapse specificity of LTP (Reymann and Frey, 2007). A strong stimulation of synapse (evoking spikes in postsynaptic neuron) induces translation of plasticity-related proteins (PRPs) which are essential for long-term maintenance of LTP and memory. At the same time, the stimulation modifies the synapses by creating hypothetical “tags” which ensure that some of the PRPs generated in the cell body will be captured only at the stimulated (activated) synapses. Because proteolysis, in contrast to other posttranslational protein modifications (such as e.g., phosphorylation), is irreversible, it is a good candidate for a synaptic tag. This possibility seems to be supported by the observation that, in most cases, inhibitors or genetic knockout of plasticity-related proteases impair LTP maintenance. The initial evidence in support of this hypothesis came from elegant *in vitro* electrophysiological studies showing neuropsin-dependent synaptic tagging and cross-tagging in the CA1 stratum oriens (Ishikawa et al., 2011). However, the idea that synaptic tagging could be related to activity-dependent extracellular proteolysis only started to be investigated. Strong mossy fiber input to CA3 may prime synaptic plasticity in upstream CA3-CA3 synapses in a process similar to a cross-tagging (Kobayashi and Poo, 2004). Since burst activity in single MF axon can generate action potentials in postsynaptic cell (Henze et al., 2002) and plays an instructive role in associative synaptic plasticity at CA3 recurrent excitatory synapses (Wojtowicz and Mozrzymas, 2014), mossy fiber LTP may drive expression of plasticity-related proteases in postsynaptic CA3 PC which is expected to affect, in turn, synaptic plasticity of the associational-commissural CA3 network.

## General Conclusions

Activity-driven structural and functional refinement of neuronal connections is thought to be a key element in mechanisms of information storage and memory formation. In this context, activity and experience-dependent extracellular proteolysis is attracting increasing attention as soluble or membrane proteases emerge as key players in controlling neuronal plasticity. It should be emphasized that the interplay between proteases and ECM proteins affects both structural and functional changes in synapses, thereby mechanistically connecting these aspects of plasticity. Indeed, in the mossy fiber pathway ECM constituents and proteases regulate long-term plasticity, experience-driven structural pre- and postsynaptic changes, synapse maturation and reversal of this process, synaptogenesis and even neurogenesis in DG (Wojcik-Stanaszek et al., 2011a). Tight regulation of MMP-9, tPA, BACE-1 and ADAMs expression is also important because their elevated activity can contribute to mossy fiber sprouting and epileptogenesis. Thus, unraveling functions of synaptic proteases and their synaptic degradomes may be of key interest far beyond of the scope of neuronal plasticity and may shed new light on investigations of clinical relevance. But still little is known about extracellular protease expression and function in hippocampal interneurons as well as about precise localization of active proteases in synaptic and perisynaptic space. Although we know much about putative, tested mainly *in vitro* substrates of particular ECM proteases, synaptic degradomes are not fully described and await systematic studies. Such a knowledge will help to understand molecular determinants involved in brain development, plasticity and memory encoding. In conclusion, extracellular perisynaptic space it is not passive or merely supportive, but it is acting in concert with ECM macromolecules and extracellular proteases potently shaping the structure and function of synapses and neuronal networks.

## Conflict of Interest Statement

The authors declare that the research was conducted in the absence of any commercial or financial relationships that could be construed as a potential conflict of interest.
